# Increases in cholecystectomy for gallstone related disease in South Africa

**DOI:** 10.1038/s41598-020-69812-3

**Published:** 2020-08-11

**Authors:** Zafar Ahmed Khan, Muhammed Uzayr Khan, Martin Brand

**Affiliations:** 1 Department of Surgery, Faculty of Health Sciences, University of the Witwatersrand, Johannesburg, South Africa; 2 Department of Surgery, Faculty of Health Sciences, University of Pretoria, Pretoria, South Africa; 3 School of Physiology, Faculty of Health Sciences, University of the Witwatersrand, Johannesburg, South Africa

**Keywords:** Cholelithiasis, Epidemiology

## Abstract

Studies suggest that the rate gallstone disease in Africa is low. Previous studies suggested an increase in gallstone rates and cholecystectomies related to urbanization and the adoption of Western lifestyle habits. This study examined cholecystectomy rates for gallstone disease in South Africa (SA). An audit of cholecystectomies in SA was done by reviewing gallbladder specimens processed by the SA National Health Laboratory Service (NHLS) from 2004 and 2014. Urbanization rates were obtained from Statistics South Africa and BMI data from previously published studies. Fisher’s exact test, *t* test’s and Pearson’s R were used for comparisons; cholecystectomy rates were calculated per 100,000 population. 33,467 cholecystectomy specimens were analysed. There was a 92% absolute increase in cholecystectomies during the study period (Pearson r 0.94; *p* < 0.01) with the overall cholecystectomy rate increasing by 65% from 8.36 to 13.81 per 100,000 population. The data was divided into two equal periods and compared. During the second period there was a 28.8% increase in the number cholecystectomies and patients were significantly younger (46.9 vs 48.2 years; *p* ≤ 0.0001). The Northern Cape was the only province to show a decline in the cholecystectomy rate in this period and was also the only province to record a decline in urbanization. Population based studies in SA demonstrate increases in BMI and an association with increased urbanization. This nationwide African study demonstrates a sustained increase in cholecystectomies for gallstone disease. Increases in BMI and urbanization may be responsible for this trend.

## Introduction

Gallbladder disease is a common and costly pathology. The development of gallstones varies among population groups around the world^1^. In developed societies the rate of gallstone disease averages between 10 and 15%. The risk of developing symptomatic disease is approximately 2–3% per year and 10% five years after the development of stones^1^. There is a causal association between changes in dietary intake, increases in body mass index (BMI) and symptomatic cholelithiasis^2^.

In the last thirty years the burden of disease has increased by more that 20% in the United States^1^. In the paediatric population this increase has been attributed to increases in BMI^3^. Population based studies have also identified an increased BMI as a risk factor for subsequent cholecystectomy^4^. As a result the number of procedures for gallstone disease has risen with cholecystectomy becoming the most common elective surgical procedure in the United States^1^.

Two South African epidemiological studies completed during the 1980′s suggested that the rate of gallstone disease in the black “urban” African population was low^5,6^. It was noted, however, that there was an increasing trend of patients with gallstone disease and the number of cholecystectomies being performed^5,6^. An increasing exposure to a Western lifestyle was cited as the potential causal association.

We hypothesized that the cholecystectomy rate for gallstone related disease has increased in South Africa. This may be as a result of increases in BMI and the effects of urbanization on BMI resulting in an increase in gallstone disease and thus cholecystectomy rates for gallstone related disease in South Africa.

## Methods

### Data source

The South African National Health Laboratory Service (NHLS) was established by legislation in 2001, enabling the amalgamation of all laboratories in the public health service. The public sector hospitals service 82.5% of the South African population and thus provide a cross sectional study of the South African population^7^. Paid medical insurance allows for access to private healthcare in South Africa for 17.5% of the population, with resulting cholecystectomy specimens processed in private pathology laboratories and are not included in this study. Therefore the population under examination consists of 87% black Africans, 8.5% mixed race, 2.6% white and 1.7% Indians^8^. Patient ethnicity is no longer recorded on NHLS pathology request forms. It is standard practice for all cholecystectomy specimens to be sent for histological analysis in South Africa. Cholecystectomy data was obtained from the NHLS prospective histological database for the period 2004–2014. South Africa has nine provinces and data was collected and categorised according to the province in which the cholecystectomy was performed. Kwa-Zulu Natal province was excluded for comparative statistics due to data only being available after 2010. In 2014 Kwa-Zulu Natal had a population of 10 million people accounting for 19.6% of the South African population^7^. Therefore our analysis is likely to represent approximately 66% of the total South African population. The data obtained was analysed for errors and duplicates were removed. Data on urbanization were obtained from Statistics South Africa. Ethics approval for the study was granted by the University of Witwatersrand Human Research Ethics Committee and the NHLS (Reference M140935). Due to the retrospective nature of the study informed consent was not obtainable. All data was retrieved independently by the NHLS database manager and all patient identifiers removed before being transferred to the study authors. Data analysis was done in accordance to regulations set out by the WITS HREC and NHLS.

### Inclusion and exclusion criteria

All patients over 12 years of age with cholecystectomies for gallstone related disease were included in the analysis. Any cholecystectomy performed during the course of another operation and where gallstone related disease was not the cause of the primary pathology were excluded (e.g. liver surgery, biliary bypass procedures, pancreatic surgery, and trauma).

### Statistics

The objectives of this study were descriptive and the database was very large, hence no sample size estimation was necessary. Descriptive analysis of the data was carried out as follows: Categorical variables were summarized by frequency and percentage tabulation. Continuous variables were summarized by the mean, standard deviation, median and interquartile range. Comparison of categorical variables were carried out using Fisher’s exact test and continuous variables using independent samples t-test. Calculation of cholecystectomy rates was per 100,000 population. This was done by excluding the 17.5% patients with medical insurance that are not treated by public service hospitals and the Kwazulu Natal population. Pearson’s correlation coefficient was used to compare the number of cholecystectomies performed per year. Medical insurance data was obtained from Statistics South Africa^7^. Data analysis was carried out using SAS version 9.4 for Windows. A 5% significance level was used.

## Results

A total of 34,294 cholecystectomy specimens were processed during the study period. Following exclusions, 33,467 were analyzed. The proportion of female patients in this series was 85% with a female to male ratio of 5.7. The mean age of the study population was 47.3 years (SD 15 years). Male patients had a mean age of 52.2 years (SD 15.2 years) and were significantly older than female patients with a mean age of 46.4 years (SD 14.7 years) (*p* < 0.0001). There was an absolute increase of 92% in the number of cholecystectomies during the study period (Pearson r 0.94; *p* < 0.01), and the cholecystectomy rate increased by 65% from 8.36 to 13.81 per 100,000 population. The highest number of cholecystectomies were performed in the two provinces that have the highest rates of urbanisation, namely Gauteng and the Western Cape provinces.

**Figure 1:**
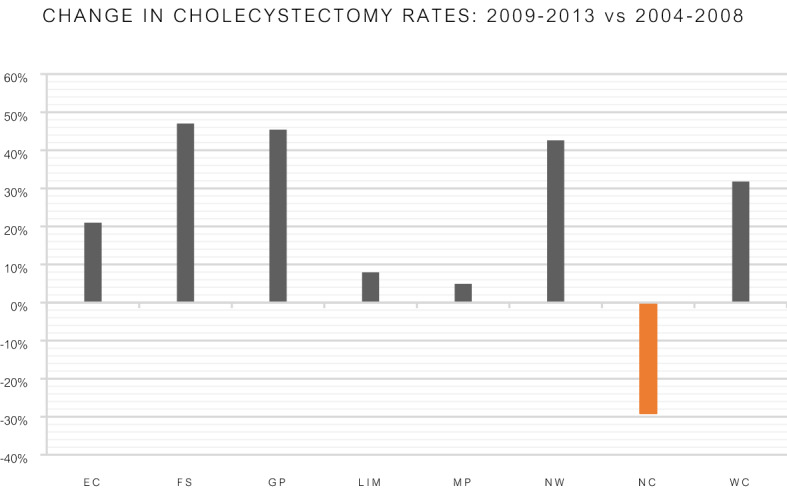
Percentage change in cholecystectomy rates in the last decade per province^9^. Adapted from Khan ZA, 2018, University of the Witwatersrand. Used with permission.

The data was divided into two equal time periods, period 1 (2004–2008) and period 2 (2009–2013). Kwa-Zulu Natal province was excluded due to data only being available after 2010. Overall there was a 28.8% increase in the number of cholecystectomies during period 2 compared to period 1 (Fig. 1). Seven of the eight studied provinces recorded increases in cholecystectomy rates. The Northern Cape was the only province to show a decrease in the cholecystectomy rate. There were 29% fewer cholecystectomies in the Northern Cape in period 2. Overall, during period 2, the patients undergoing cholecystectomy were significantly younger than during period 1 (48.2 vs 46.9 years; *p* ≤ 0.0001). This was true for both female (47.4 vs 46.1 years; *p* ≤ 0.0001) and male patients (52.6 vs 51.5 years; *p* = 0.0362). There was no significant change in the male to female ratio (*p* = 0.4156). At the beginning of the study there were only three hospitals in the country that performed more than 100 cholecystectomies in a calendar year and all were affiliated to a university or located in large metropolitan areas. At the end of period 2 fifteen hospitals were consistently performing more than 100 cholecystectomies in a year, with all but three university affiliated hospitals or in major metropolitan areas.

## Discussion

The rate of gallstone disease among women is reported to be 2–3 times higher than men^10^. Our finding of a 1:6 male to female ratio is in keeping with the literature albeit at a higher rate. Male patients were significantly older than female patients in this study which is also consistent with other cholecystectomy studies^11^. In period 2 of the study there was a significant trend for younger patients to have a cholecystectomy. This is an unusual finding as the incidence of cholelithiasis increases with age^12^, and suggests that other risk factors may be more prevalent in the study population.

**Figure 2:**
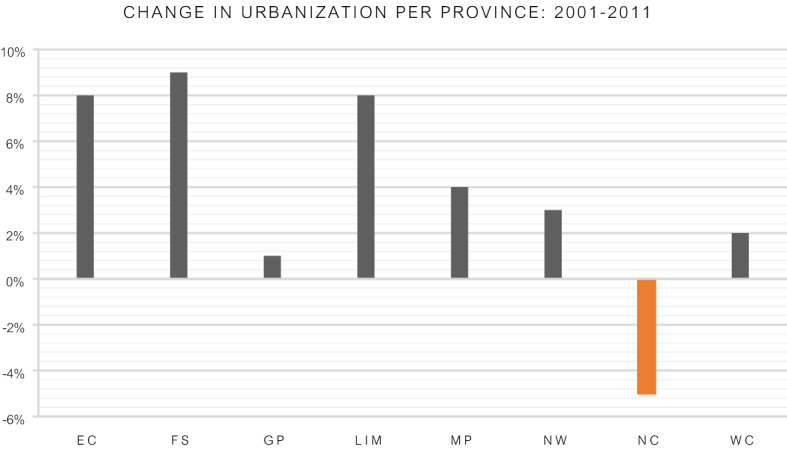
Percentage change in urbanization in the nine South African provinces^7, 9^. Adapted from Khan ZA, 2018, University of the Witwatersrand. Used with permission.

This study, the largest from Africa, demonstrates an increase in the number of cholecystectomies for gallstone related disease in South Africa over a 10-year period. Cholecystectomy studies from the early 1990′s have attributed the increased rate of cholecystectomies to a lower threshold for surgery and enthusiasm associated with the advent of less morbid and better tolerated laparoscopic surgical procedures^11,13–15^. The increases from studies on cholecystectomy has ranged from 20 to 53%^11, 13,15,16^. A continued and sustained increase in cholecystectomy rates are ascribed to changes in risk factors; specifically dietary changes and increasing body mass index^2,11,17^. South Africa has the highest prevalence of obesity in Sub-Saharan Africa with the highest incidence among black South African women^18^. A study investigating obesity trends and risks factors in South Africa was conducted between 2008 and 2012^19^. The investigators reported an increasing trend in BMI among South African adults although rural dwellers had a lower baseline BMI compared to their urban counterparts. An increase in BMI was suggested to be due to the adoption of “urban lifestyles”. Figure 2 depicts the change in the percentage of the population residing in urban and rural areas between the 2001 and 2011 census with a positive value indicating an increase in the number of people residing in urban areas and a negative value a decrease in the number residing in urban areas. Urbanization trends in South Africa over the last decade show that all but one province had an increase in rural to urban migration^8^. The Northern Cape experienced a 5% decline in urbanization. It was also the only province in our series to show a decline in the cholecystectomy rate (Fig. 1). The urbanization and BMI data lend support to previous data suggesting urbanization together with increases in BMI are associated with an increase in symptomatic cholelithiasis^5^. This together with the decreasing age of cholecystectomy patients all lend additional support to this hypothesis. A consideration regarding access to care in South Africa has to be made. Patients residing in rural areas are often disadvantaged and may not have access similar to their urban counterparts^20^. However in our study, apart from the Northern Cape, all the other provinces with a rural pre-dominant population continued to show increases in cholecystectomy rates.

Additionally, two studies conducted in Soweto, South Africa, during the 1980′s suggested that although the rate of cholelithiasis in the black “urban” African population was low, there was an increasing incidence together with increases in cholecystectomy rates^5, 6^. It was suggested that an increased exposure to a Western lifestyle, namely increased saturated fat with a decreased fiber intake, was a possible risk factor for this. A similar study from Japan also suggests that westernization of the Japanese diet after World War 2 has resulted in an increase in symptomatic gallstone disease^21^.

During the last 30 years the burden of gallstone disease has increased by more that 20% resulting in an estimated annual expenditure of approximately $6.5 billion in the United States^2^. The South African health system is plagued by four concurrent epidemics, namely infectious diseases, specifically HIV, maternal death, malnutrition and an increasing burden of other non-communicable diseases^22^. The almost doubling in the number of cholecystectomies during the last decade represents a significant increased burden on an already strained system. This data provides a platform from which the population may be educated regarding the risks of gallstone disease and the plausibility for the implementation of risk reduction strategies, specifically community education and weight loss programs.

Low hospital volume for surgical cases has been associated with morbidity and mortality^23^. Several studies exploring cholecystectomy outcomes in relation to hospital volume suggest that high volume centers are associated with reduced morbidity and costs^24,25^. However, in patients with average operative risk, the clinical outcomes are not different between low and high volume centers^26^. In South Africa, most high-risk patients are referred to tertiary hospitals. This study suggests that most tertiary institutions are now high-volume centers and would be able to adequately manage these patients, thus reducing morbidity and costs.

This study has several limitations. Clinicodemographic data obtained was limited to that provided by surgeon and pathologist. A second limitation may be related to increases in cholecystectomy rates after the introduction of laparoscopic cholecystectomy. Our findings of a 92% increase in the cholecystectomy rate are higher than international trends and thus cannot be accounted for simply by the introduction of laparoscopy alone. Furthermore, the introduction of laparoscopic cholecystectomy in South Africa occurred in the early 1990′s, more than 10 years prior to this study. Although the increase in cholecystectomy rates may suggest an increase in the prevalence of symptomatic cholelithiasis, cholecystectomy rates alone are subject to several sources of bias and may therefore be a crude indication of the prevalence of gallstone disease. These include variations in surgical rates as suggested above and variations over time^27^. Several methods including ultrasound, autopsy studies and oral cholecystography have been used to estimate prevalence with all having their specific drawbacks^27^. Although it is practice for all cholecystectomies done at public hospitals are submitted to the NHLS, there is a possibility that some may not have been sent. Furthermore, although the NHLS provided all of the electronic data available, there may be gaps in the system as some data may have been logged manually and not inserted electronically into the database. An estimated number of these cases was not available. The large number of patients included in this study may mitigate against the incomplete data.

In conclusion we have demonstrated through analysis of 33,467 gallbladder specimens for gallstone related disease in South Africa over a 10-year period that the number of cholecystectomies has increased significantly. The results of this study suggest a change in the disease pattern in South Africa. Increases in BMI together with increases in urbanization appear to be a significant risk factor in the aetiology of gallstone disease. This is particularly worrisome as the population under study has historically been regarded as being at low risk for the development of gallstone disease. The increasing trend in cholecystectomies has implications of increased cost and burden of disease and is thus likely to become a public health problem in a system that is already plagued by other concurrent epidemics (infectious disease, malnutrition).
